# The Paradox of Availability: A Quantitative Analysis of Veterinary Medicinal Products for Aquaculture in Europe Between 2018 and 2026

**DOI:** 10.3390/vetsci13070713

**Published:** 2026-07-20

**Authors:** Lauren Chambers, Wiebke Jansen, Dušan Palić, Andrea Fabris, Alain Schonbrodt, Leona Nepejchalová, Nancy De Briyne

**Affiliations:** 1 Federation of Veterinarians of Europe, 1030 Brussels, Belgium; 2 Faculty of Veterinary Medicine, Ludwig-Maximilians-University Munich, Kaulbachstr 37, 80539 Munich, Germany; d.palic@lmu.de; 3 Italian Association of Fish Farmers (API), Via Del Perlar 37/A, 37135 Verona, Italy; 4 Professional Veterinary Union (UPV), Rue Des Frères Grisleins 11, 1400 Nivelles, Belgium; 5 Institute for State Control of Veterinary Biopreparations and Pharmaceuticals (ÚSKVBL), Hudcova 232/56a, 621 00 Brno, Czech Republic

**Keywords:** aquaculture, fish health, limited markets, market consolidation, regulation (EU) 2019/6, regulation (EU) 2019/4, Union Product Database (UPD), Veterinary Medicinal Products (VMPs)

## Abstract

Availability of veterinary medicinal products is a critical determinant of health, welfare, and economic sustainability in European aquaculture. This study assessed the changes in the authorization and reported market availability of veterinary medicines for finfish in the European Union (EU) and European Free Trade Association (EFTA) between 2018 and 2026, by comparing and analyzing data on veterinary medicinal products for aquatic species from the Heads of Medicines Agencies (HMA), the FishMedPlus Coalition, and the European Medicines Agency Union Product Database. The results revealed a wide divergence between regulatory authorization and reported availability. While the total number of authorized VMPs nominally increased from 304 in 2018 to 332 in 2025 (+9.2%), the number of reported unique active substances available declined (−16.7%). Crucially, the analysis of 2025 data indicates that only 26.5% of authorized products were reported to be actively available on the market, creating a “paper market” that masks severe therapeutic gaps, particularly in minor fish species such as seabass, seabream, carp, and trout sectors. Potential key drivers identified were limited markets, industry consolidation, increased burden related to stricter environmental requirements, as well as enlarged administrative and financial burdens. Despite EU legislative mechanisms designed to support limited markets, the sector faces stagnation in therapeutic diversity, necessitating continued reliance on the off-label use of medicines.

## Simple Summary

Availability of veterinary medicinal products (VMPs) is a critical determinant of health, welfare, and economic sustainability in European aquaculture. This study assessed the development in the authorization and reported market availability of veterinary medicines for finfish in the European Union and European Free Trade Association between 2018 and 2026. Drawing on data from the Heads of Medicines Agencies, the FishMedPlus Coalition, and the European Medicines Agency Union Product Database, the results revealed a divergence between regulatory authorization and reported availability. While the total number of authorized VMPs nominally increased from 304 in 2018 to 332 in 2025, the number of reported authorized VMPs with unique active substances available declined from 84 to 70. Crucially, an analysis of current data indicates that only 26.5% of authorized products are reported to be actively marketed, creating a “paper market” that masks severe therapeutic gaps, particularly in seabass, seabream, carp, and trout sectors. Key drivers identified were limited markets, industry consolidation, increased environmental requirements, and the unintended consequences thereof. Despite legislative mechanisms designed to support limited markets in the European veterinary medicines legislation, the sector faces stagnation in therapeutic diversity, necessitating continued reliance on the off-label use of medicines.

## 1. Introduction

Aquaculture is a strategic sector for the European Union (EU), contributing significantly to food security and the “Blue Economy” [1]. This is evidenced, in part, by the fact that the European Commission is working to establish a 15-year strategic framework, Vision 2040, with the goal of achieving long-term sustainability and competitiveness of the EU fisheries and aquaculture sector [2]. However, the sector’s sustainable growth is among other issues hampered by the limited availability of authorized veterinary medicinal products (VMPs) [1]. Unlike terrestrial livestock sectors, aquaculture involves an even higher diversity of species, each with their own disease particularities. Many species are reared in open husbandry systems with direct or close contact with the surrounding environment, creating unique challenges for the aquatic ecosystems, which necessitate an environmental risk assessment procedure before VMP market authorization is granted [3]. The lack of available authorized VMPs not only compromises fish health and welfare but also obliges veterinarians to rely on a special provision for this situation, allowing veterinarians in exceptional circumstances to use VMPs authorized for a different aquatic or terrestrial food-producing animal, humans, or to be prepared extemporaneously in order to avoid unacceptable suffering. However, this approach, known as the cascade and laid down in Art. 114 (3) Regulation (EU) 2019/6, also creates legal and logistical uncertainties and can mask the real lack of VMP availability [4]. Availability was defined, as suggested previously by Čudina et al., 2026, as a product which is both marketed and authorized, at either a national or EU level; notably, this means there may be a shortage of available products, for example, due to supply chain disruptions [5].

Historically, the market for fish VMPs has been considered a “minor use/minor species” (MUMS) or “limited market” by the pharmaceutical industry and, within policy, was characterized by high regulatory costs and low returns on investment [6]. In 2019, the EU overhauled its veterinary pharmaceutical legislation with Regulation (EU) 2019/6 on VMPs and Regulation (EU) 2019/4 on medicated feed, which became fully applicable in January 2022 [4,7]. Regulation (EU) 2019/6 aimed to reduce administrative burdens, improve the functioning of the internal market, increase availability of products, reduce antimicrobial resistance, and stimulate innovation through specific incentives for limited markets (Article 23). It also established a single publicly available VMP database, the Union Product Database (UPD), whose public interface is the Veterinary Medicine Information Website [4,8]. Regulation (EU) 2019/4 aimed to ensure the safe, responsible, and traceable use of VMPs in animal feed across the EU [7]. Delegating and implementing acts supplement the two Regulations, of which several have a profound further impact on the manufacturing, use, and availability of VMPs for finfish [9].

In 2019, the FishMedPlus coalition, a joint initiative by researchers, published a gap analysis of VMP availability within the aquaculture sector [10], identifying barriers to improvement and proposing corresponding solutions. Through these actions, recommendations were made to facilitate greater VMP availability in the aquaculture sector. For example:

• Authorization of new medicines or vaccines through stimulating the animal health industry to invest more into the research and development of finfish VMPs, including vaccines;
• Extending the marketing authorization of already authorized products between countries (Mutual Recognition Procedure (MRP), or the further procedure described in Reg. (EU) 2019/6 as the Subsequent Recognition Procedure (SRP));
• Extending existing marketing authorizations to other species via former line extensions (currently as the variations requiring assessment Chapter I. of the guidance for explanation of the practice of Art. 60 to 68 of Regulation (EU) 2019/6) [11];
• Stimulating further industry investments.

By comparing baseline data from the VMPs listed for the main aquaculture species (specifically food-producing finfish aquaculture) in 2018 with current data on authorizations and availability, this article aims to analyze whether the VMP availability in European aquaculture has improved, while evaluating the impact of regulatory changes and market dynamics from 2018 to 2026.

## 2. Materials and Methods

This study integrates quantitative regulatory data with qualitative policy analysis to assess changes in VMP authorization and availability for the main aquaculture fish species (specifically food-producing finfish aquaculture, VMPs for ornamental fish were out of the scope). The geographic scope encompassed the EU-27 and two EFTA countries (Norway and Iceland), with particular focus for the main species within six of the major aquaculture producers: Germany, Greece, Italy, Norway, Poland and Spain, capturing the wide range of real vs. listed availability in these countries.

**Data Sources**: For 2018, regulatory data on authorized VMPs for fish were obtained from an Excel dataset provided by the Coordination Group for Mutual Recognition and Decentralized Procedures—Veterinary (CMDv) of the Heads of Medicines Agencies (HMA) (Supplementary Materials Table S1).

For the current situation, two datasets were used. Firstly, a UPD extracted dataset (Excel sheets per finfish species) of December 2025 (Supplementary Materials Table S2), which was complemented and verified by UPD searches in January and February 2026. In order to realistically represent practical gaps experienced by practitioners in the field, the VMPs with the same active substance(s) were grouped together (e.g., oxytetracycline hydrochloride authorized in the same country/species by different marketing authorization holders (MAHs)). Both datasets included key attributes such as trade name, active substance(s), authorization status, target species, ActVet Code, and marketing authorization holders. In the 2025 and 2026 datasets, a reported availability status field was present, while in the 2018 dataset, it was unavailable.

**Desktop Research and Expert Solicitation**: Complementary information was collected from reports relevant to aquaculture and veterinary regulation, including those produced by the FVE FishMedPlus Coalition, the Federation of European Aquaculture Producers (FEAP), the European Advisory Commission on Aquaculture (Aquaculture Advisory Council, AAC), and reports from the EMA pertaining to aquaculture [10,12,13,14]. To contextualize and extend the regulatory data and comparison between the listing of 2018 versus 2026, the authors reviewed the product authorization and availability in their countries to identify gaps and unmet needs.

**Definitions Used**: Definitions of gaps and unmet needs reflect those used in the VMP Gaps and Needs Compass for Sheep and Goats [5].

## 3. Limitations

This study relied primarily on 2018 data from the CMDv and the Union Product Database in 2026. While these sources provide comprehensive information on VMPs across the EU, several limitations should be noted. First, for the year 2018, data on reported product availability was not available, preventing direct comparisons with later years. Second, the availability status recorded for 2026 in the UPD relied on the reporting by marketing authorization holders (Article 58(5) of reg. (EU) 2019/6; Article 18(5) of reg. (EU) 2021/16) [4,15] and may not have been updated. Expert feedback indicated discrepancies in the databases: some products listed as available in a given country were, in practice, not actually accessible, whereas other products reported as unavailable were in fact obtainable. Therefore, reported product availability may not always reflect actual market availability due to delays or inconsistencies in UPD or national database updates. These inconsistencies may have introduced errors in the assessment of temporal trends and selected cross-country comparisons per species. Also, the comparisons did not include consideration of weighting by production volume. Consequently, the findings should be interpreted with caution, particularly regarding the reported availabilities of specific products. Despite these limitations, this study provides valuable insights into the evolution and current status of VMPs available for fish in the EU.

## 4. Results

### 4.1. Comparison of Authorized Fish VMPs in 2018 Versus 2025

A quantitative comparison of the VMPs available for the food-producing finfish aquaculture landscape revealed a paradoxical trend. In absolute terms, the number of marketing authorizations (MAs) for fish VMPs appeared to increase over time. In 2018, a total of *n* = 304 fish VMPs were authorized across the reviewed countries, including *n* = 28 authorized in the United Kingdom (UK), which withdrew from the European Union on 1 February 2020. By December 2025, the total number of authorized fish VMPs had risen to *n* = 332, representing an increase of 9.2% (+28 authorizations). While the UK accounted for 28 authorized fish VMPs in 2018, Northern Ireland remained included in the 2025 and 2026 analysis because it continues to apply certain EUVMP rules under the post-Brexit regulatory arrangements. In 2026, Northern Ireland recorded 19 authorized fish VMPs.

**Figure 1:**
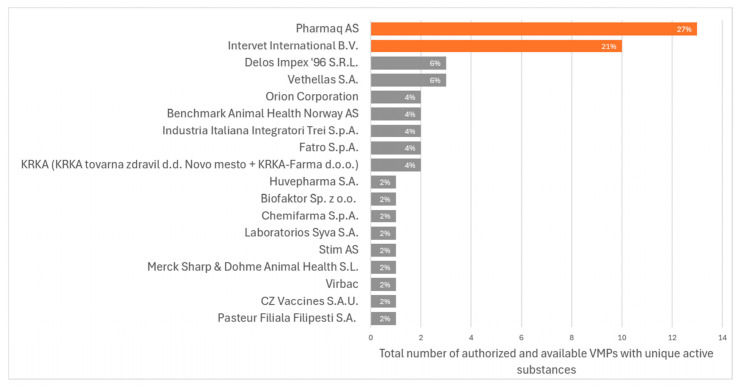
Per marketing authorization holder, total number of authorized and available VMPs with unique active substances.

Looking however, at unique active substances, in 2018, there were 84 unique active substances authorized; in December 2025, there were 70 (see Table 1). So, while the number of authorized products increased, the number of active substances declined. It was also noted that the majority of authorized VMPs were held by 2 MAHs (see Figure 1).

### 4.2. Comparison of Authorized Versus Reported Available Products

The new feature introduced in 2024 provided by the Union Product Database (UPD) allows for the calculation of an “availability rate.” Availability was defined as a VMP reported to be “Available” in the UPD, meaning a product is actually marketed/supplied in that Member State, i.e., not only listed as authorized but genuinely commercially available for ordering/use. In December 2025, of the *n* = 332 VMPs holding a valid marketing authorization, *n* = 88 VMPs (26.5%) were reported to be ‘available’ in the UPD. Following further UPD searches, the number of authorized and available products in February 2026 varied per country (see Table 2). Further UPD searches also allowed comparison of per species availability, compared to 2018 (see Table 3).

## 5. Discussion

The European finfish market is characterized as large in tons of protein output; however, the corresponding veterinary medicinal products (VMP) market remains relatively small and fragmented. Further development of this market may be necessary to support the continued growth of aquaculture as a sustainable source of proteins.

The total European production of fish by aquaculture is estimated to be 2,898,188 tons in 2024 [16]. Norway was the dominant producer in Europe with 55% of the total supply, consisting mainly of salmon, but also with significant trout production. The other countries that produce more than 100,000 tons of finfish in Europe annually are Turkey, United Kingdom, and Greece. The main species produced are salmon, trout, seabream, seabass, and carp, which together represent 92% of the total European production in 2024 [16].

The FishMedPlus Coalition was composed of many organizations and institutions active in the European aquaculture sector, chaired by FVE and comprising 10 experts spanning the aquaculture industry, academia, regulatory authorities, legislators, the animal health industry, and the veterinary profession—Animal Health Europe, FEAP, World Aquatic Veterinary Medical Association (WAVMA), University of Stirling, EAFP, Ludwig-Maximilians University Munich (LMU), Faculty of Fisheries and Protection of Waters University of South Bohemia (FFPW USB), and Institute for State Control of Veterinary Biopreparations and Pharmaceuticals (ÚSKVBL) (CZ). In 2018, worried about the lack of VMPs to treat fish, the coalition produced a gap list for salmon, trout, seabass, seabream, and carp of the main priority diseases/indications for which treatment options were urgently needed [17]. This list covered:

• Ectoparasites—Ich (Ichthyophthirius or Ichthyophthiriosis), costia (Ichthyobodosis), sea/salmon lice, *Monogenea* infestation—all species;
• Bacterial diseases—*Aeromonas* spp.—all species;
• Fungal and Oomycotic infections—all species;
• Amoebic gill disease (AGD)—mostly salmon;
• Rainbow Trout Fry syndrome (RTFS)—Flavobacteriosis—trout and carp;
• Sedation and anesthesia—all species;
• Viral diseases—all species (see WOAH list);
• Hormones for broodstock management/maturation/ovulation induction;
• Endoparasites—all species.

Following identification of these gaps, the FishMedPlus Coalition identified hypothesized pertinent barriers and worked on solutions for having more aquatic VMPs authorized and available. Potential solutions identified were: 1/ authorization of new VMPs through stimulating the animal health industry to invest more into research and development of fish VMPs and vaccines, 2/ extending the marketing authorization of already authorized VMPs between countries (Mutual Recognition Procedure (MRP) or Subsequent Recognition Procedure (SRP)), 3/ extending existing marketing authorizations to other species via line extensions (currently as the variations requiring assessment Chapter I. of the guidance for explanation of the practice of Art. 60 to 68 of Regulation (EU) 2019/6), and 4/ stimulating industry to invest via regulatory incentives (lower data requirements, longer data protection time, etc.) [18].

### 5.1. Limited Market with Many ‘Ghost Licenses’, Dominated by Authorizations for Antimicrobials

The diversity of farmed fish species in aquaculture, each with distinct biological characteristics, husbandry systems, and disease profiles, can create unique challenges that differ substantially from those encountered in terrestrial livestock production. As a result, the EU aquaculture sector remains comparatively small and fragmented. This limited market size may contribute to a reduced commercial incentive for pharmaceutical companies to invest in the development, registration, and maintenance of VMPs for aquatic species. From an industry perspective, investing in products intended for a relatively restricted and highly specialized market may be considered economically less attractive. These dynamics may explain why the aquaculture sector is characterized by a limited number of pharmaceutical companies actively developing finfish VMPs, as well as a comparatively low availability of authorized and commercially accessible VMPs for fish species within the EU.

To give some examples: Poland, being the largest producer of carp in the European Union, with an annual carp production of typically between about 17,000 and 21,500 tonnes of fish per year, only has one product reported as authorized and available for carp (including when incorporating wider ‘fish’ umbrella categories), namely, antimicrobials: oxytetracycline hydrochloride [19,20]. No VMPs authorized and available for the treatment of parasitic or viral diseases, or for sedation, were identified for carp in Poland through the UPD search. Another problem is how often products are temporarily unavailable, creating ‘shortages’ on the market. Whilst Belgian practitioners can make use of the ‘cascade’ system, importing VMPs from other countries is further complicated by the small size of the domestic market. Belgium, whilst not being a main finfish producer, nevertheless has some trout and carp production, and has no available finfish.

In addition, a limited number of fish VMPs are authorized through the centralized procedure, which would allow their use across all EU Member States, provided the marketing authorization holder (MAH) chooses to market them in those countries. Due to the costs involved in this procedure, this approach may not be feasible in cases where sales volumes are low. The vast majority of finfish VMPs instead hold national authorizations and are therefore only authorized in one or a limited number of European countries, which may further restrict product availability across the EU.

This hypothesized limited market attractiveness is further reflected in the discrepancy observed between the number of authorized VMPs and the number of products that are genuinely available on the market. Analysis of the UPD revealed that nearly three-quarters of the VMPs authorized for use in European aquaculture are not commercially marketed and effectively exist only as so-called “ghost licenses” within regulatory databases. While these products formally retain a marketing authorization, they are not accessible in practice to veterinarians or fish producers. The high number of ghost licenses is not unique to fish VMPs but is an ongoing problem more widely in veterinary medicine [21]. A similar availability rate was recently found for VMPs for small ruminants in the EU [5]. It should be noted that marketing authorization holders (MAHs) are responsible for maintaining up-to-date information regarding the status and availability of authorized products. Nevertheless, the (co-)authors saw some discrepancies with the ‘availability status’ in the UPD and the situation in their country. For example, for Germany, the information in Table 2 was cross checked with two German databases listing VMPs availability only for formalin preparations and sodium chloride as authorized VMPs for fish; search strings used were “fish”, “trout”, “carp”, “aquaculture”, “salmon”, and “aquatic” (original queries were in German) [22,23]. No available hormones, antimicrobials, vaccines, or other substances were listed. The issue may be due to a lag or a gap in updating these databases, as some listed VMPs go as far back as 1961. Such inconsistencies may lead to inaccurate assessments of medicine availability and consequently affect evidence-based policymaking.

Another hypothesized driver identified was that a few VMPs not listed as authorized and available on UPD, were in fact identified by the (co-)authors as being available in their country. One example was praziquantel: while it is listed as unavailable in the UPD, Italian experts confirmed that it is on the market in Italy and Greece.

Unfortunately, the 2018 listings did not include information on market availability; so, it was not possible to assess how this phenomenon has evolved over time. Nevertheless, it may be hypothesized that the number of “ghost licenses” has unintentionally increased following the removal of the “sunset clause”. Under Directive 2001/82/EC, marketing authorizations would automatically lapse if a product was not placed on the market within a specified period, generally three years. The removal of this provision may therefore have allowed inactive authorizations to remain indefinitely within regulatory databases despite products no longer being commercially available. In addition, market consolidation within the animal health sector may have further contributed to this trend.

This high number of ‘ghost licenses’ has many negative effects. Most importantly, it makes important products not available to treat finfish. This lack of availability of important VMPs for fish affects animal health, welfare, and productivity. In addition, it may lead policymakers to believe that VMP coverage is adequate because authorizations exist, possibly creating an misunderstanding of the actual situation, resulting in under-prioritization of research or incentives for new products and potentially discouraging new entrants or potential competitors.

Looking at the classes of authorized VMPs, the predominance of antibiotics for finfish aquaculture remains a concern in the context of antimicrobial resistance (AMR), as noted by several recent publications [19,20], even if actual number of substances is lower compared to other animal production systems [24]. Prioritization of preventive health strategies, particularly vaccination, may help to reduce the need for antimicrobial treatments and support more sustainable aquaculture production. The Norwegian salmon sector provides an example of the efficacy of this approach: through the widespread implementation of vaccination programs, combined with improved biosecurity and finfish welfare measures, antibiotic use in salmon farming has been reduced by more than 99% since the late 1980s, while production volumes have continued to increase substantially [25].

Expanding the development, availability, and uptake of alternatives to antibiotics, such as effective vaccines, including autogenous vaccines where appropriate, should therefore be considered a potential key pillar of sustainable aquaculture and AMR reduction strategies [19,20].

### 5.2. Considerable Market Consolidation Happened Between 2018 and 2026

Our results showed that the veterinary pharmaceutical sector has undergone significant consolidation. In 2018, 47 unique companies held MAs for fish products. By 2025, this number dropped to 40, but, more tellingly, only 19 companies had products actively available on the market (Table 4), and only two companies (Pharmaq and Intervet International) are responsible for roughly one-half of the products on the EU and EFTA markets.

Since 2018, two major consolidations have happened. Following the earlier acquisition of Pharmaq, Zoetis consolidated its dominance in the vaccine and diagnostic space by acquiring the Fish Vet Group in 2020 [26], and, in 2024, MSD Animal Health acquired the entire aquaculture business of Elanco for $1.3 billion [27]. This last merger consolidated two of the largest portfolios, bringing key products like Clynav (DNA vaccine) and Imvixa (lufenuron) under a single entity. Imvixa, however, despite having an MRL for fish, is not authorized in the EU or Norway for fish. While this creates a “one-stop-shop” for salmon producers, it raises concerns about reduced competitive pressure to develop niche products for limited markets.

It remains to be elucidated if market consolidation within the animal health sector may have further contributed to the trend of “ghost licenses”. Mergers and acquisitions can result in companies retaining authorizations for legacy products without actively manufacturing or marketing them, thereby increasing the number of authorized but unavailable VMPs listed for aquaculture species.

The consolidation observed in the aquaculture pharmaceutical sector reflected a broader trend across the veterinary medicines industry. Over the past decade, a series of landmark events reduced the number of pharmaceutical competitors in the EU livestock market, leaving five companies accounting for the majority of global veterinary pharmaceutical revenue [28]. In addition, major players are increasingly redirecting investment away from traditional VMPs for food-producing animals toward companion animal therapeutics, precision medicines, diagnostics, and digital health tools [29]. This commercial reorientation, likely driven by higher profit margins in companion animal markets, risks further marginalizing limited markets such as aquaculture, where the return on investment for new product development is already constrained by small patient populations and complex regulatory pathways [30]. The aquaculture sector, also, faces an additional structural disadvantage: unlike livestock, it lacks a generic competitor base to partially offset the reduced innovation incentive that market concentration may create.

### 5.3. New Medicine Regulations: Efforts Made to Increase Availability for Limited Markets—Progress and Remaining Challenges and Its Effect

One of the four main objectives of Regulation (EU) 2019/6 was to increase the availability of VMPs. As such, regulators introduced specific incentives for limited markets (fish are included), such as simplified data requirements, incentivizing centralized authorizations, facilitating a single market (e.g., via the UPD), introducing a specific, clearer cascade and listing mechanisms for aquatic species where authorized products are lacking (Art 114 of Regulation 2019/6 on ‘Use of medicinal products for food-producing aquatic species’) [4,31].

Of the aforementioned efforts created through Regulation (EU) 2019/6, it could be said that two of the significant ones were the facilitation of a single market through establishment of the UPD (Article 55), as well as the development of a customized cascade for aquatic animals (Article 114). The intended aims of these measures are to allow veterinarians to easily check product authorization and availability in other countries which match self-identified gaps they have experienced in their own country for certain species and indications. The Aquaculture Advisory Council (AAC, the advisory stakeholder body for the European Commission) provided expert feedback on this point. According to the AAC, it has become easier to identify authorized and available fish VMPs in other EU MS. However, practically acquiring these identified VMPs remains difficult due to the variability of import conditions and restrictions in force in different Member States [32].

Regulation (EU) 2019/6 is expected to be complemented by an implementing act (Art 114 (3)), which will list substances used in VMPs authorized for food-producing terrestrial animals, or substances contained in medicinal products for human use authorized in the Union, or substances used for extemporaneous preparations, which may be used in food-producing aquatic species in accordance with Article 114(1). This implementing act will be drafted based on the EMA scientific advice and is expected to be published in 2026 [33]. Despite these regulatory incentives created, our analysis suggests that the efforts made by the Regulation may have been insufficient.

The other new Regulation introduced was Regulation (EU) 2019/4 on medicated feed. For certain medications, e.g., when treating bacterial infections with antibiotics, especially in the grow-out production phase, it is highly impractical and unsuitable to treat each fish individually. Group treatments via the oral delivery route thus remain an essential and standard method for delivering antimicrobials and antiparasitics in aquaculture. Ideally, this is done through medicated feed, as it allows for less environmental pollution (medicines washing away in the water). Regulation (EU) 2019/4 allows the use of medicated feed in aquaculture, and the Commission Delegated Regulation (EU) 2024/1159 (‘oral medication act’) allows a special derogation for antimicrobials that recognizes the benefit of administering medication through feed (as water medication is impractical for fish) [34]. However, in reality, many medicated feed manufacturers are hesitant to produce medicated feed for fish due to the small amounts/units needed, and due to the risk of cross-contamination. While ‘on-farm mixing’ is allowed at the EU level by Regulation (EU) 2024/1159, many Member States currently limit, oppose, or prohibit such practices in their national regulations.

Looking at which new products came into force since 2018, there were two new authorizations for vaccines intended for use in salmon, as well as the (re)introduction of the substance Imidacloprid, through the establishment of a revised maximum residue limit (MRLs) for imidacloprid in finfish. An extension of MRL was adopted for praziquantel in finfish. In 2025, EMA also gave a positive opinion on Hemosyvet (etamsylate), a hemostatic (anti-bleeding) medicine for many species including seabream; however, this medicine is only used for other species (dogs, cats, etc.) and is not authorized and of necessity for finfish [35].

Despite these additional products authorized, there remains a widespread lack of available authorized VMPs for fish, thereby increasing the reliance of veterinary practitioners on the use of the “cascade” in daily practice (article 114 of Regulation (EU) 2019/6). While the “cascade” is often presented as an essential mechanism and pragmatic solution to address a lack of availability, it cannot be viewed as a sustainable long-term solution. For example, when the VMPs are used under the cascade mechanism, they are not commonly tested in, or otherwise adapted to the receiving species, and the withdrawal periods are applied using a safety margin. In principle, cascade use is only allowed under specific exceptional conditions to avoid ”unacceptable animal suffering” [36] and under the responsibility of the prescribing veterinarian. When using the cascade mechanisms, the veterinarian therefore assumes responsibility and documents the rationale for the selection of the product, justification of its use, decision on the dose and duration, all under conditions where only limited species-specific data are available. Continued reliance on the cascade may therefore contribute to variability in clinical practice between countries and does not replace the need for a broader availability of appropriately authorized VMPs for finfish.

To improve veterinarians’ ability to prescribe treatments with greater certainty regarding their safe and effective use in fish, they need to be confident in the appropriate withdrawal periods. To support this, specialized (veterinary research) institutions could collaborate with fish veterinarians and producers and publish methodologies and studies supporting recommendations of safe cascade treatment options. This is one example of how the alternative solutions (system) operate(s) in the Czech Republic (and in some other countries) under conditions of VMP unavailability. However, inconsistencies of harmonization between MS further increases restrictions in the cascade approach.

Despite these new entrants, looking at the FishMedPlus Gap list (Table 5), it remains clear that many of the most impactful diseases in fish remain severely limited in or completely without treatment options, especially without utilizing the cascade.

### 5.4. Recommendations to Increase Future Availability of Fish VMPs

Based on the findings of this study, several recommendations can be made with the aim of improving the availability of VMPs for fish in EU and EFTA countries:

• Finalization of the Article 114(3)-implementing act should be prioritized, as it would immediately expand the legal basis for cascade prescribing with greater safety certainty, reducing the burden and liability on individual prescribing veterinarians.
• Specialized veterinary research institutions, in collaboration with fish veterinarians and producers, could develop and publish evidence-based guidance on the use of treatment options under the cascade, including robust methodologies for determining appropriate withdrawal periods, to ensure more consistent and scientifically grounded clinical practice across Member States.
• The incentives introduced under Regulation (EU) 2019/6 for limited markets have proven insufficient and should be strengthened. Measures to strengthen incentives for limited markets could include fee waivers for centralized procedure applications, public co-funding of regulatory dossiers for priority indications identified in the FishMedPlus gap list, and extended market exclusivity periods, analogous to orphan drug incentives in human medicine. Possible funding at EU level may be explored in a format similar to the AADAP/FDA program in the U.S [37,38].
• Establishment of a functional single market for fish VMPs, through enhanced free circulation of authorized medicines and vaccines across Member States, streamlined Mutual Recognition Procedure, and the development of workable import frameworks for VMPs authorized in EFTA countries and relevant third countries such as the United States and Canada.
• National implementation of Regulation (EU) 2019/4 on medicated feed should be harmonized. On-farm mixing is currently permitted at EU level under Regulation (EU) 2024/1159 but remains restricted or prohibited in several Member States, effectively removing a critical and environmentally preferable administration route for aquaculture treatments.
• Future efforts should prioritize innovation in non-antibiotic disease control methods, such as vaccine development. Autogenous vaccines play a critical role in aquaculture by enabling rapid and tailored responses to emerging and region-specific pathogens, and regulators could support their responsible use through clearer harmonized rules, streamlined approval pathways, and promoting innovation. Further GMP standards for autogenous vaccines are currently under development; these standards would benefit from inclusion of good manufacturing practice for autogenous veterinary vaccines and supporting the use of products across Member States.
• MAHs should update UPD in a timely manner and report shortages when they occur. A user-reporting mechanism may be integrated into the UPD to enable veterinarians and producers to flag inaccurate or outdated availability information. A real-time, bi-directional verification mechanism such as this could increase marketing authorization holder accountability, support continuous validation of the UPD data, and ensure the UPD better reflects current national situations, thereby strengthening its value as a clinical and regulatory tool.
• Conducting focus groups to gather veterinary field perspectives on experiences/perceived lack of availability, including regulators, industry and other stakeholders, such as through the AAC and with input of professional associations such as FVE, FEAP, European College of Aquatic Animal Health (ECAAH), and WAVMA.
• Public research funding under programs such as Horizon Europe and the European Maritime, Fisheries, and Aquaculture Fund should explicitly prioritize the unmet indications identified in the FishMedPlus gap list—particularly ectoparasites, amoebic gill disease, fungal infections, and sedation—to lower the pre-competitive R&D barrier and stimulate industry investment in fish VMPs.

## 6. Conclusions

Compared with terrestrial animals, availability of VMPs for use in food-producing aquatic animals is extremely limited in number [39,40,41]. Only a few products, mainly antibacterials and antiviral vaccines, are available, and in many cases, the products are only authorized in a few countries. Looking at the increasing demand for fish products, the high import of fish from outside the EU, and the stable fisheries sector, it is clear that there is a strong role that European aquaculture can play in filling the gap between EU consumption and domestic supply. To increase the EU’s self-sufficiency level, European aquaculture needs to grow substantially. Therefore, the sector urgently needs improvements in access to authorized VMPs, especially alternatives to antibiotics, to prevent and treat the most common diseases and secure animal welfare. This study shows the most important therapeutic gaps in the main finfish species farmed in Europe, while making several recommendations to improve the availability of VMPs for fish in the EU and EFTA countries.

## Figures and Tables

**Table 1:** Comparison of authorized finfish veterinary medicinal products (VMPs) in the EU and EFTA, 2018 versus 2025.

Metric	2018 EU + EFTA Status of Authorized Finfish VMPs	2025 EU + EFTA Status of Authorized Finfish VMPs	Change (%)
Total Authorizations	304	332	+9.2%
Authorizations for Unique Active Substances *	84	70	−16.7%

Source: Derived from HMA 2018 listings and UPD extracts from December 2025. * This data reflects only authorized products, not availability. Lack of 2018 availability data prevented direct comparison of availability figures to quantify change.

**Table 2:** Authorized and available finfish veterinary medicinal products (VMPs) by country and main species, with calculated availability rates.

	Column 1	Column 2	Column 3	Column 4	
Country and Species *	2026 ** Authorized VMPs	2026 ** Authorized Distinct VMPs with Unique Active Substances	2026 ** Authorized and Reported Available Distinct VMPs with Unique Active Substances	Availability Rate based on Total Authorized VMPs (Column 3/Column 1)	Market Characteristics
Norway—Atlantic Salmon (including Salmonid)	31	20	17	55% (17/31)	High availability of vaccines (9 distinct), anesthetics (6 authorized and available, comprising 3 unique active substances) and sea lice treatments (4 unique authorized and available products).
Greece—Seabass and/or Seabream	18	14	8	44% (8/18)	3 unique antibiotic substance available (oxytetracycline hydrochloride; oxolinic acid; TMPS), 4 distinct vaccines authorized and available (all for seabass, 1 also authorized for trout) No anesthetics authorized, 1 parasiticide (praziquantel).
Italy—Trout (including Salmonid) and/or Seabass	39	30	9	23% (9/39)	5 authorized and available unique active substance antibiotics (Florfenicol; chlortetracycline; oxytetracycline; flumequine; TMPS), 3 authorized and available unique vaccines for seabass, one hormone (Buserelin acetate). Low availability—especially for trout—despite high authorization count.
Poland—Carp	1	1	1	100% (1/1)	Extremely low product diversity; only 1 antibiotic authorized for carp (oxytetracycline).
Spain—Seabass	9	8	2	22% (2/9)	Only 2 products authorized and available (vaccines for seabass). Extremely low availability.
Germany—Trout (including Salmonid) and/or Carp	11	10	1	9% (1/11)	Only 1 vaccine authorized and available for trout (against *Yersinia ruckeri*). No antimicrobials, antiparasitics or analgesics are authorized and available.

* Search filters included only what is listed in this column, with addition of ‘fish’ category. ** Checked on UPD search between December 2025 and February 2026.

**Table 3:** Authorized, available, and distinct finfish veterinary medicinal products (VMPs) by species across EU and EFTA countries.

Species	Total Authorized VMPs 2026 *	Total Authorized and Available VMPs 2026 *	Total Authorized, Available and Distinct VMPs (with Unique Active Substances) 2026 *^,^**	Number of VMPs with New Active Substances Newly Authorized and Available Between 2018 and 2026
Atlantic Salmon (including overarching ‘Salmonid’ category)	47	27	20 (Multiple vaccines; Deltamethrin; Isoeugenol; Imidacloprid; Benzocaine; Oxytetracycline; TMPS; Tricaine mecylate; Bronopol; Azamethiphos; Emamectin benzoate)	3 (2 new vaccines available; Imidacloprid)
Seabass	17	8	5 (4 Vaccines and Oxytetracycline)	0
Seabream	6	2	1 (2 oxytetracycline products are authorized and available under 2 different companies)	0
Trout (including overarching ‘Salmonid’ category)	56	25	14 (2 vaccines; Deltamethrin; Florfenicol; Isoeugenol; Benzocaine; Imidacloprid; Oxytetracycline; TMPS; Tricaine mesylate; Flumequine; Bronopol; Buserelin; Emamectin benzoate)	1 (Imidacloprid)
Carp	17	6	2 (Oxytetracycline; Florfenicol)	0
Any products for ‘Fish’/‘Other Fish’/Fish (fresh water) which were not already included in species-specific numbers above	49	15	8 (oxytetracycline; florfenicol; chlortetracycline; flumequine; enrofloxacin; oxolinic acid; tricaine mesylate; TMPS)	0

Number of authorized VMPs, authorized and available VMPs, and authorized/available VMPs with unique active substances, by species, across selected EU and EFTA countries (Austria, Belgium, Bulgaria, Croatia, Cyprus, Czechia, Denmark, Estonia, Finland, France, Germany, Greece, Hungary, Iceland, Ireland, Italy, Latvia, Liechtenstein, Lithuania, Luxembourg, Malta, Netherlands, Norway, Poland, Portugal, Romania, Slovakia, Slovenia, Spain, Sweden, and the United Kingdom (Northern Ireland)). * Checked via UPD search between December 2025 and February 2026. ** Distinct here is defined as containing a unique active substance.

**Table 4:** Animal health companies active in the field of aquaculture in EU and EFTA in 2018 and 2026.

Metric	2018: Number of Companies with Authorized Products	2026: Number of Companies with Authorized Products *^,^**	2026: Number of Companies with Authorized and Available Products *^,^**	Change (2018 compared to 2026 in Number of Unique Companies and Continuing vs. Left)
Total unique companies	47	40	19	−7
Companies continuing		21		46.8% of 2018
Companies that left	26			55.3% of 2018
New companies		19		45% of 2026

* These results do not represent the number of unique products per company, UPD search provides overlaps with the same active substance(s) for different products, and same products with separate authorizations held in different countries. ** Reviewed via UPD search February 2026.

**Table 5:** Authorized and available finfish veterinary medicinal products (VMPs) in 2026 compared with needs gaps identified in 2018 by the FishMedPlus Coalition, by species across EU and EFTA countries; (**a**) Atlantic salmon (including overarching ‘Salmonid’ category); (**b**) seabass and seabream; (**c**) trout (including products authorized for all fish); (**d**) carp.

(**a**) Atlantic Salmon		
Needs Gaps * Identified in 2018, in Order of Importance: Were They Filled or Do They Remain as an Unmet National or Unmet European Need	Number of VMPs with New Active Substances Newly Authorized and Available Between 2018 and 2026	2026 Update ** of Needs Gaps Identified in 2018 (New API Authorized Increase = Green Box, Decrease = Orange Box or No Change = Transparent Box)
Ectoparasite infections	2 new vaccines available (ALPHA JECT micro 7 ILA i) (Alpha Ject Moritella, emulsion for injection for Atlantic salmon); Imidacloprid.	Ectoparasite infections: improvement via introduction of imidacloprid; hydrogen peroxide available 2018, is still authorized, but reported unavailable 2026; Teflubenzuron available in 2018 but not 2026.
Viral infections		Viral infections: improvement due to 2 new vaccines including coverage of infectious salmon anemia virus and infectious pancreatic necrosis virus (coverage for infectious salmon anemia virus is new, coverage for infectious pancreatic necrosis virus was already available in 2018). Authorization for ALPHA JECT Micro 1 Noda (against red-spotted Grouper Nervous Necrosis Virus (RGNNV)) removed for salmon between 2018 and 2026.
Fungal infections		Fungal infections: no change
Amoebic Gill Disease (AGD)		Amoebic Gill Disease (AGD): no change
Tapeworms		Tapeworms: no change
Other parasites		Other parasites: no change
(**b**) Seabass and Seabream		
Needs gaps * identified in 2018, in order of importance: were they filled or do they remain as an Unmet National or Unmet European need.	Number of VMPs with new active substances newly authorized and available between 2018 and 2026	2026 update ** of needs gaps identified in 2018 (New API authorized Increase = green box, Decrease = orange box or No Change = transparent box)
Endoparasite infections: 0 VMPs	Praziquantel (MRL extrapolation)	Endoparasite infections: increase by 1 VMP
Ectoparasite infections: 0 VMPs		Ectoparasite infections: no change
Bacterial infections: 11 treatment options (among which several vaccines for seabass/seabream)		Bacterial infections: reduced to 9 VMPs
Viral infections: one vaccine against RGNNV in seabass		Viral infections: no change
(**c**) Trout		
Needs gaps * identified in 2018, in order of importance: were they filled or do they remain as an Unmet National or Unmet European need.	Number of VMPs with new active substances newly authorized and available between 2018 and 2026	2026 update ** of needs gaps identified in 2018 (New API authorized Increase = green box, Decrease = orange box or No Change = transparent box)
Ectoparasite infections 2 treatment options	1 (Imidacloprid)	Ectoparasite infections: improvement via introduction of imidacloprid (from 2 to 3 VMPs)
Bacterial infections (9 VMPs authorized, most for ‘all fish’)		Bacterial infections: Decrease in number of antibiotics authorized (reduced from 9 to 8 VMPs)
Viral infections: 2 VMPs		Viral infections: Decrease in number of VMPs (only 1 remaining)
Parasitic myxozoan infections: 0 VMPs		Parasitic myxozoan infections: no change
Fungal and oomycotic infections: 1 VMP		Fungal and oomycotic infections: no change
Amoebic Gill Disease (AGD): 0 VMPs		Amoebic Gill Disease (AGD): no change
Other (Red Mark Disease, Puffy Skin disease, Strawberry disease, Rainbow Trout Gastroenteritis): 0 VMPs		Other (Red Mark Disease, Puffy Skin disease, Strawberry disease, Rainbow Trout Gastroenteritis): no change
Other: 5 VMPs (anesthetics—hormones)		Other: decrease by one hormonal VMP
(**d**) Carp		
Needs gaps * identified in 2018, in order of importance: were they filled or do they remain as an Unmet National or Unmet European need.	Number of VMPs with new active substances newly authorized and available between 2018 and 2026	2026 update ** of needs gaps identified in 2018 (New API authorized Increase = green box, Decrease = orange box or No Change = transparent box)
Ectoparasitic infections: 0 VMPs	0	Ectoparasitic infections: no change
Fungal and oomycotic infections: 0 VMPs		Fungal and oomycotic infections: no change
Bacterial infections: (6 VMPs authorized, most for ‘all fish’)		Bacterial infections: no change
Viral infections: 0 VMPs		Viral infections: no change
Other (Carp edema virus, Koi sleeping disease, etc): 2 hormonal VMPs		Other (Carp edema virus, Koi sleeping disease): decrease by one hormonal VMP
Sedation and anesthesia: 0 VMPs		Sedation and anesthesia: no change

* Definitions of unmet needs mirrors Veterinary Medicines Gaps and Needs Compass for Sheep and Goats [5]. ** Checked on UPD search between December 2025 and February 2026.

## Data Availability

The original contributions presented in this study are included in the Supplementary Materials. Further inquiries can be directed to the corresponding author.

## Supplementary Materials

The following supporting information can be downloaded at: https://www.mdpi.com/article/10.3390/vetsci13070713/s1, Table S1: list of VMPs in 2028; Table S2: list of VMPs in 2025.
